# Interplay between Cytoskeletal Stresses and Cell Adaptation under Chronic Flow

**DOI:** 10.1371/journal.pone.0044167

**Published:** 2012-09-19

**Authors:** Deepika Verma, Nannan Ye, Fanjie Meng, Frederick Sachs, Jason Rahimzadeh, Susan Z. Hua

**Affiliations:** 1 Department of Physiology and Biophysics, SUNY-Buffalo, Buffalo, New York, United States of America; 2 Department of Mechanical and Aerospace Engineering, SUNY-Buffalo, Buffalo, New York, United States of America; West Virginia University, United States of America

## Abstract

Using stress sensitive FRET sensors we have measured cytoskeletal stresses in α-actinin and the associated reorganization of the actin cytoskeleton in cells subjected to chronic shear stress. We show that long-term shear stress reduces the average actinin stress and this effect is reversible with removal of flow. The flow-induced changes in cytoskeletal stresses are found to be dynamic, involving a transient decrease in stress (phase-I), a short-term increase (3–6 min) (Phase-II), followed by a longer-term decrease that reaches a minimum in ∼20 min (Phase-III), before saturating. These changes are accompanied by reorganization of the actin cytoskeleton from parallel F-actin bundles to peripheral bundles. Blocking mechanosensitive ion channels (MSCs) with Gd^3+^ and GsMTx4 (a specific inhibitor) eliminated the changes in cytoskeletal stress and the corresponding actin reorganization, indicating that Ca^2+^ permeable MSCs participate in the signaling cascades. This study shows that shear stress induced cell adaptation is mediated via MSCs.

## Introduction

Renal epithelial cells experience a wide range of shear stress due to urinary flow. Through various adaptation mechanisms cells adjust their cytoskeleton structure, adhesion assembly and cell-cell interactions, thereby accommodating the mechanical challenge [1], [2]. This remodeling minimizes local stresses and affects membrane transport and other cell functions [3], [4]. Exposure of renal tubule cells to chronic flow results in reorganization of the cytoskeleton and an increase in the formation of cell-cell junctions [5], [6]. While the molecular mechanisms of shear stress transduction remain unclear, flow shear stress appears to induce conformational changes in a variety of cytoskeleton proteins [7]. These, in turn, can activate biochemical pathways that affect cell morphology, migration and growth [8], [9], [10].

The spatial and time-dependent distribution of external force applied to the cells is inherently non-uniform because the cytoskeleton is a heterogeneous and anisotropic collection of dynamically cross-linked proteins. Stress varies with the intrinsic elasticity of individual proteins, the dynamics of cross-links, and the degree of prestress [11], [12], [13], [14], [15]. The adaptation of cells to mechanical stimuli occurs over multiple time scales [16]. At short time scales, external force induces rapid and reversible changes in cytoskeletal stresses associated with elastic stretching of load-bearing proteins [8], [16]. This is followed by conformational changes of the proteins [7]. With longer term stimulation, the cytoskeleton undergoes chronic rearrangements [14], [17], [18], [19].

The response of cells to short term (∼seconds) mechanical stimuli has been studied by various means [20], [21], [22], [23]. Applying force to integrin receptors by a pipette or using ligand-coated magnetic beads attached to the cell surface causes cell stiffening via changes in focal adhesion assembly at the stimulation sites [20], [24]. This increase in stiffness can extend to periods lasting minutes [16]. We have previously shown that a pulsatile shear stress results in reversible changes in stress within α-actinin, and these changes decay gradually with multiple challenges. Thus the adaptation begins at time scale of seconds (<45 sec) [25]. Although cells may use multiple sensors to activate adaptation mechanisms, the earliest adaptation response appears to be consistently towards increased stiffness. However, epithelial cells in the kidney experience chronic (long term) fluid shear stress (in the range of minutes to hours) and experience a large variation in mechanical forces. The dynamics of adaptation to flow involves polymerization and depolymerization of F-actin, changes in cross linking, and correlated changes in migration and growth [26], [27]. With the development of our genetically coded force sensitive fluorescent probes [28], [29], we are able to directly measure the variation of stress in specific proteins and visualize the accompanying changes in cytoskeleton structure in real time and begin pinpointing which proteins are involved in adaptation.

In this study, we have measured actinin stresses and simultaneously observed corresponding cytoskeletal anatomy in Madin Darbey Canine Kidney (MDCK) cells subjected to chronic fluid shear stress, using the stress sensitive FRET sensor referred to as spectrin repeat stress sensitive FRET (sstFRET) [29]. Our study reveals that long-term (∼3 hrs) exposure to fluid shear stress produces three distinct phases of cytoskeletal stress variation, each lasting for minutes. This process was inhibited in the presence of Gd^3+^ and GsMTx4, blockers of mechanosensitive ion channels (MSCs) [30], [31], [32], [33].

## Results

### Co-localization of actinin-sstFRET with F-actin in MDCK cells

**Figure 1:**
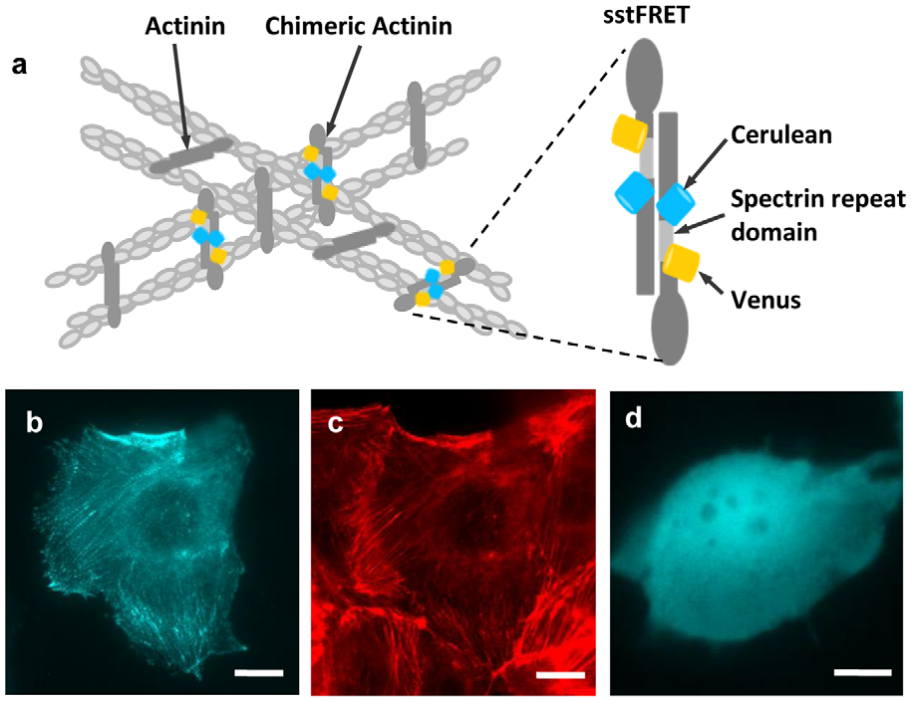
Actinin-sstFRET sensor construction and expression in MDCK cells. **a:** Schematic of actinin-sstFRET sensors inserted in actin cytoskeleton. The sensor consists of Cerulean as donor, Venus as acceptor, and a linker (spectrin repeat domain), and is inserted close to the middle of actinin. **b:** Fluorescence image of actinin-sstFRET in MDCK cells. **c:** Fluorescence image of F-actin stained with phalloidin-Alexa Fluor 568, showing co-localization of actinin-sstFRET with actin. **d:** Fluorescence image of MDCK cells expressing free sstFRET, showing uniformly distributed fluorescence molecules in cytosol. The scale bars represent 10 µm.

We expressed the FRET probe, actinin-sstFRET (Fig. 1a), in MDCK cells and then subjected the cells to a constant flow in a microfluidic chamber. The sensitivity of this FRET sensor has been previously characterized using DNA springs in solution and is on the order of 5 pN. The sensor was also expressed in cells and it does not affect physiological function of the host protein [29] (see Materials and Methods section for further details on the FRET stress sensor). Actinin-sstFRET co-localizes with F-actin, which allows us to use the FRET probe as a label for F-actin to monitor actin translocation. Colocalization is shown by expressing actinin-sstFRET in MDCK cells (Fig. 1b) and subsequently staining actin with phalloidin-Alexa Fluor 568 (Fig. 1c). As a control, we also transfected the free sstFRET cassette itself, i.e., without its linkage to actinin, and the fluorescence image in Fig. 1d shows that free probe is uniformly distributed in the cytosol (Fig. 1d).

### Dynamics of cytoskeletal stresses under chronic flow

**Figure 2:**
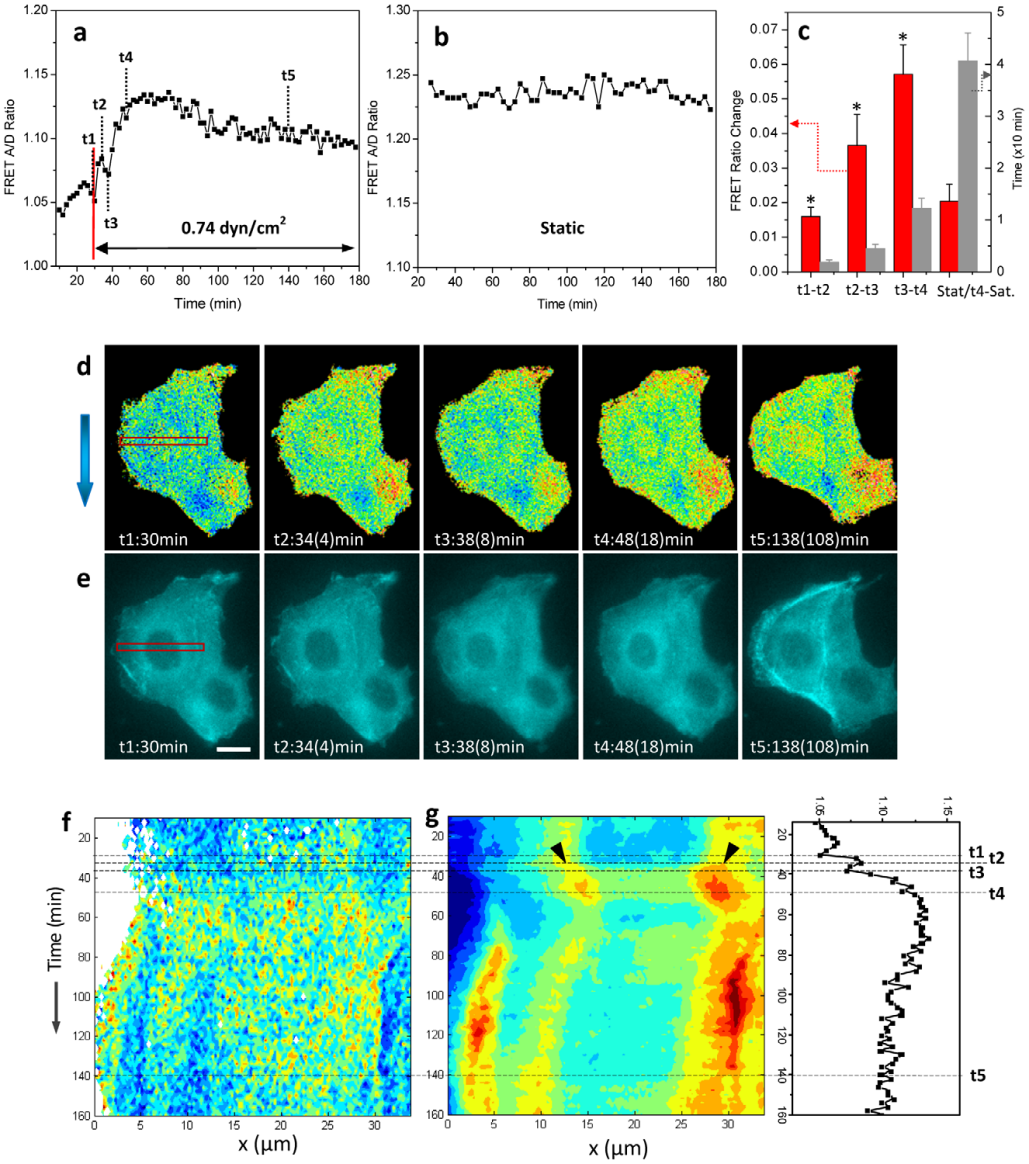
Shear stress adaptation and actin reorganization. **a:** Time dependent change of average FRET ratio in the upper cell in (**d** and **e**), showing that changes in actinin stress occur in three distinct phases under shear stress of 0.74 dyn/cm^2^. **b:** Typical variation of average FRET ratio for cell under static condition in the chamber. **c:** Statistics of FRET peak changes for three phases (n = 7) and variation under static conditions (n = 8) (red, left axis); and statistics of time period for three phases and to saturation (n = 7) (gray, right axis). (*) indicates peaks observed under flow. **d,e:** Respective FRET ratio and CFP images of two cells subjected to shear stress at times indicated in (a). The FRET ratio is shown in 16 color map, where blue indicates a low FRET ratio and higher tension; red indicates a higher FRET ratio and lower tension. The numbers in brackets indicate the duration under flow. The arrow on the left of (d) indicates flow direction. The scale bar represents 10 µm. **f, g:** Time dependent FRET distribution along the window in (d), and time dependent actin intensity along the window in (e). These distributions are correlated with various phases in (a), which is also replotted for ease of discussion.

We followed changes in stress in cells subjected to a steady shear flow (0.74 dyn/cm^2^) for up to 3 hrs and found that the average stress decreased and then stabilized. Figure 2a shows time dependent changes in the FRET ratio averaged over an entire cell under flow; the (red) line marks the start of flow. Remarkably, we consistently observed three distinct phases. In Phase-I, marked t1–t2 in Fig. 2a, there was a rapid increase in FRET ratio (a decrease in stress) lasting 1–2 min. This was followed by a decrease in FRET (an increase in stress) that took ∼3–6 min to reach a minimum (Phase-II, t2–t3). This was followed by a long term (∼15 min) increase in FRET (decrease in stress) (Phase-III; t3–t4). Following this, the FRET ratio stabilized or slightly decayed under the continuous flow lasting up to 3 hrs (t>t4). These dynamics were consistently observed in repeated experiments (n>10) and the statistics of the peak FRET ratio change for each Phase (left axis) and the time period (right axis) for three phases are shown in (Fig. 2c). In contrast, for cells under no-flow condition, the FRET ratio varied only slightly with time (Fig. 2b) (its statistics are also shown in Fig. 2c).

Corresponding to the various phases shown in Fig. 2a, Figs. 2d,e show the simultaneously acquired FRET images (Fig. 2d, movie S2) and the underlying reorganization of F-actin (Fig. 2e, movie S1). The image in Fig. 2e corresponding to Phase-I (Fig. 2e, t1–t2) did not show significant actin redistribution indicating that the decrease in stress at this stage is due to the deformation of actinin proteins themselves; this is consistent with our previous results [25]. The short-term increase in stress during Phase-II is associated with an inward contraction of F-actin. It is characterized by a shrinking nucleus and an increase in the density of F-actin around the nucleus (Fig. 2e, t2–t3). No such ‘ring formation’ around the nucleus was observed in cells under a static environment (movie S3, S4). Phase III, which was accompanied by a large decrease in stress, was found to be associated with an observable dissociation of actin, Fig. 2e (t3–t4). Longer times following t4 are characterized by actin reassembly, resulting in F-actin bundles at the cell perimeter (Fig. 2e, t5). Although the above results are shown for a shear stress of 0.74 dyn/cm^2^, cells responded similarly to stresses as low as 0.3 dyn/cm^2^ but the magnitude of responses was reduced (data not shown).

The spatiotemporal changes in cytoskeletal stress is further analyzed by mapping time-dependent changes in FRET ratio and F-actin distribution across a given region of interest (red windows in Figs. 2d,e), and this analysis is shown in Fig. 2f and 2g, respectively. (For convenience, the FRET ratio in Fig. 2a is also shown juxtaposed with Fig. 2f,g). As seen in Fig. 2g, there is no significant change in F-actin density or redistribution over the course of phase-I (t1–t2), representing the earlier stage of deformation of actinin proteins. A decrease in FRET ratio (increase of stress) corresponding to phase-II (t2–t3) is seen in Fig. 2f as a color change from warm (red/yellow) to cool (blue/green); the accompanying inward movement of enhanced actin density is marked by the black arrows in Fig. 2g indicating contraction of the actin network around the nucleus by the end of phase-II. The correlated increase in stress and actin contractile motion suggests that the cytoskeletal tension is responsible for the increase in F-actin concentration around the nucleus. Notice that the dense actin ring persists in Phase-III (t3–t4, Fig. 2g), however, the mean cytoskeletal stress decreases to a minimum during this time (Fig. 2f). This observation suggests that contraction triggers F-actin disassociation in cells. At t5, there is a high actin density at the cell periphery (Fig. 2g) marking actin reassembly under flow shear stress.

**Figure 3:**
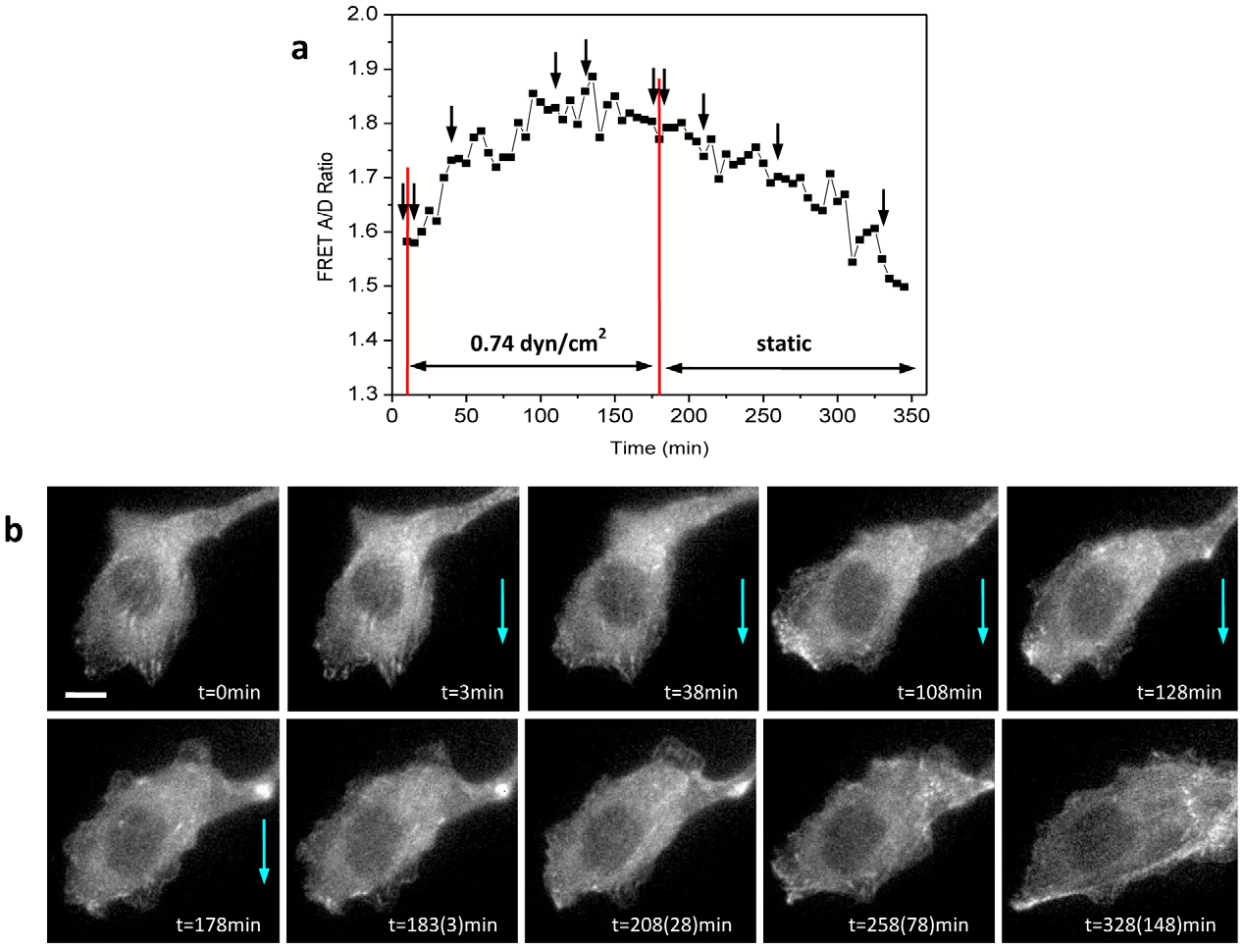
Reversible cytoskeleton reorganization under fluid shear stress. **a:** Changes of average FRET ratio in a cell subjected to shear stress of 0.74 dyn/cm^2^ for 3 hrs and subsequently in stop-flow condition, showing actinin softening in flow and hardening in the no-flow condition. The changes in flow are indicated by red lines. **b:** Fluorescence images (CFP channel) at times indicated in (a), showing a reversible F-actin reorganization. The scale bar represents 10 µm.

We showed that flow-induced cytoskeleton reorganization is a reversible process at short times [25]. To examine whether reorganization can be reversed at longer times, we applied a steady flow (0.74 dyn/cm^2^) for 3 hrs and then stopped the flow. By measuring the changes in stresses and the structure we found that cytoskeleton reorganization is also reversible over a time scale of hours. Figure 3a shows the average stress in a cell measured as the FRET ratio and Fig. 3b shows the simultaneously recorded F-actin structure. Figure 3a shows that flow causes an increase in FRET ratio that returns reversibly to its initial state upon removal of flow. The corresponding images in Fig. 3b show that under flow (blue arrows) the actin fibers gradually disappear, but they reappear after the flow is stopped. It shows that the flow induced softening and correlated cytoskeleton reorganization is reversible; the parallel F-actin bundles were restored (Fig. 3b) and the cells returned to a state of higher stress (lower FRET) when the flow was stopped (Fig. 3a).

### Inhibition of mechanosensitive channels diminishes cytoskeleton reorganization

**Figure 4:**
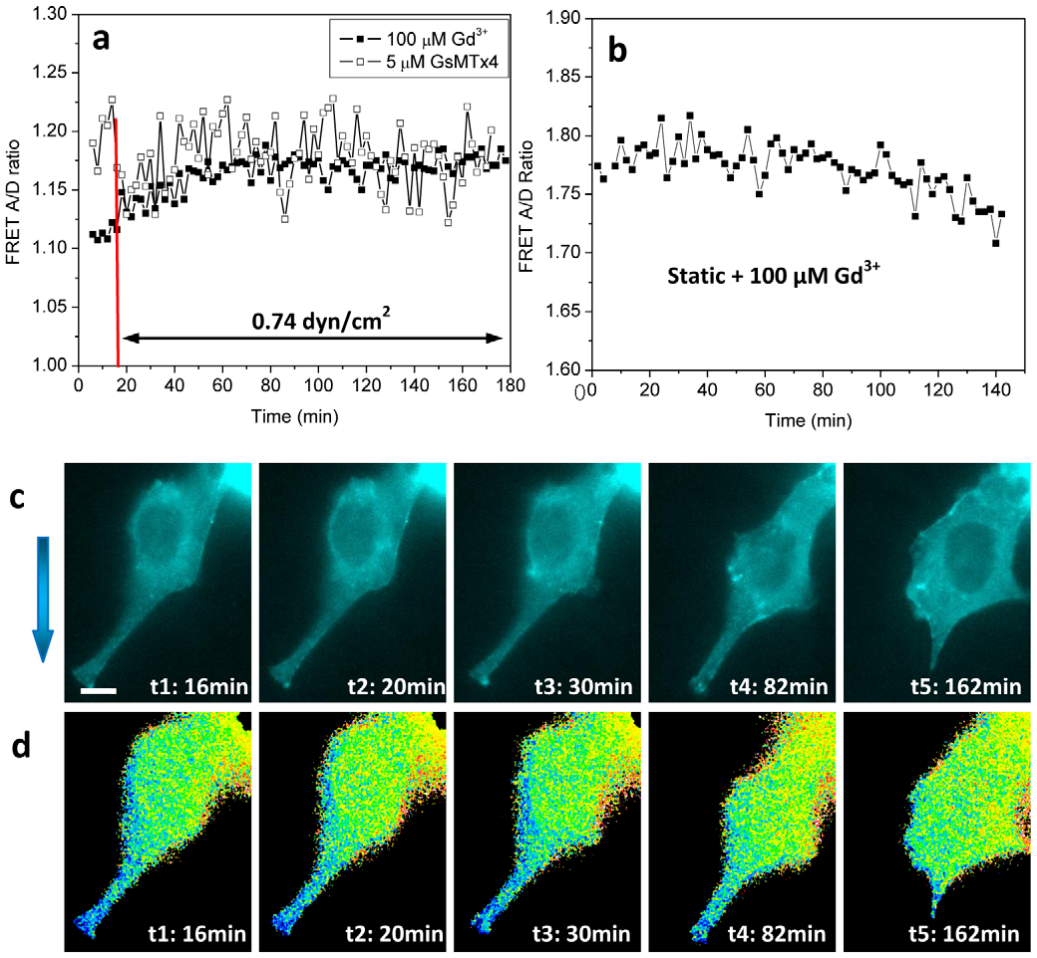
Fluid shear stress-induced cytoskeletal stresses in the presence of MSC inhibitors Gd^3+^ and GsMTx4. **a,b:** Application of 100 µM Gd^3+^ (solid squares) or 5 µM GsMTx4 (unfilled squares) inhibited the dynamic changes in actinin stresses under flow, showing only small variations. **c,d:** Fluorescence images (CFP channel) and FRET ratio at various times in (a) (solid square), showing that shear stress did not cause significant redistribution of cytoskeleton, and cell showed active locomotion. The scale bar represents 10 µm.

Previous studies have suggested that shear stress may activate mechanosensitive channels (MSCs) and trigger Ca^2+^ entry that affects contractile forces and cytoskeleton structure [34], [35]. To examine the role of MSCs we measured the average FRET ratio under flow, with and without MSC blockers: 100 µM Gd^3+^ or 5 µM GsMTx4, the latter being the only known specific blocker of MSCs. The shear stress-induced reduction in cytoskeletal stresses was blocked by both Gd^3+^ and GsMTx4 (Fig. 4a). Under static condition the inhibitors had no obvious effects on the cells (Fig. 4b). Figures 4c,d show the CFP images and FRET ratio, respectively, of the cell in flow condition in the presence of Gd^3+^ at various times (Fig. 4a, also see movie S5, S6). Under shear there were no distinct phases of stress variation (Fig. 4a) or any associated F-actin accumulation around the nucleus in the presence of MSC inhibitors. We have also observed that shear stress of 1 dyn/cm^2^ caused Ca^2+^ increase in MDCK cells and the response can be blocked by the same amount of MSC inhibitors (Fig. S1), suggesting the involvement of Ca^2+^ permeable MSCs in the remodeling.

**Figure 5:**
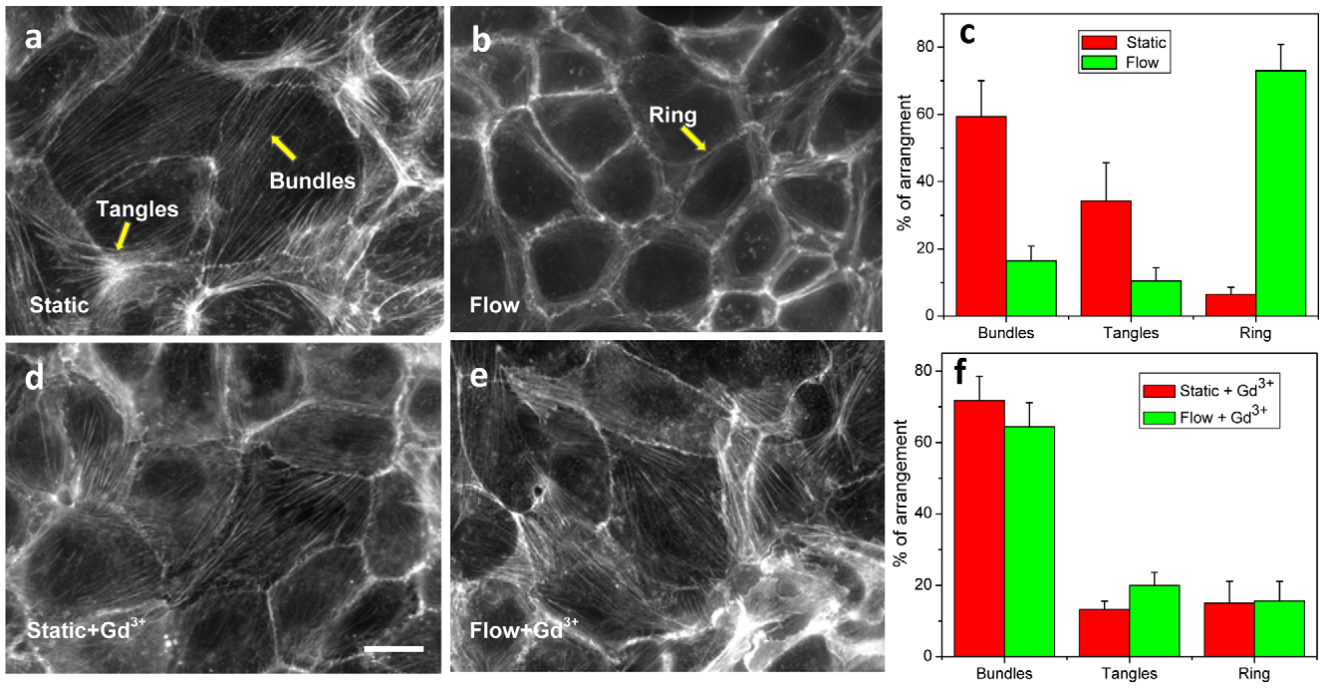
Effect of mechanosensitive channel inhibitors on flow-induced cytoskeleton reorganization. Immunostained F-actin in MDCK cells under **a:** static condition and **b:** shear stress of 0.74 dyn/cm^2^ for 3 hrs. **c:** Statistics of actin arrangements under static (red) and shear stress (green). The percentage was estimated for each experiment, and the error bars show standard errors of “n” experiments (static, n = 7; shear flow, n = 10). **d:** F-actin staining in the presence of 100 µM Gd^3+^ in cells in static condition and **e:** under shear flow of 0.74 dyn/cm^2^ for 3 hrs, showing Gd^3+^ inhibits flow induced cytoskeleton reorganization. **f:** Statistics of actin arrangements of (d) vs (e) (static+Gd^3+^: n = 4; flow+Gd^3+^: n = 8). The scale bar is 20 µm.

To examine actin itself we stained F-actin using phalloidin in fixed cells. Figure 5a,b shows cells with no flow versus those subjected to a shear stress of 0.74 dyn/cm^2^ for 3 hrs, respectively. They clearly show that actin bundles are randomly distributed throughout the cells cultured under static condition (Fig. 5a). In contrast, Fig. 5b shows that shear stress rearranges the F-actin bundles to the cell periphery. This flow-induced F-actin reorganization was abolished in the presence of 100 µM Gd^3+^. This is shown in Fig. 5d,e where abundant F-actin can be seen in the cytosol without and with flow, respectively, and the results were consistent across the experiments (n = 7) (Fig. 5c,f). Thus MSC-mediated signaling is involved in the reorganization of the cytoskeleton induced by shear stress.

### Shear stress reduces cell mobility via MSC dependent pathway

**Figure 6:**
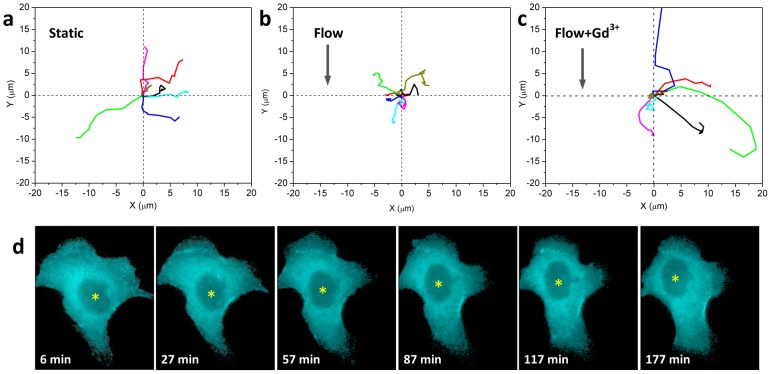
Effect of shear stress on cell motility. **a–c:** Trace of single cell movement tracked by centroid of the nucleus for 2.5 hrs under **a:** static condition; **b:** shear stress of 0.74 dyn/cm^2^; and **c:** same flow condition with addition of 100 µM Gd^3+^. The cell's original location (defined by the center of nucleus) is at (0,0) and the motion is tracked along the x and y axis every 16 min for a total ∼160 min. The arrows in **b** and **c** indicate flow directions. **d:** CFP images showing the typical movement of a cell under static condition. The yellow stars in (d) indicate the center position of nucleus that was traced and plotted in (a). The scale bar represents 10 µm.

To determine whether the F-actin rearrangement induced by shear stress affects mobility, we analyzed cell movement with a 2D map of the nucleus location with and without flow. Under static (no flow) conditions the cells were motile (Fig. 6a), and the time lapse images (Fig. 6d) show typical movements. A shear stress of 0.74 dyn/cm^2^ markedly reduced the cell motility (Fig. 6b) and adding 100 µM Gd^3+^ lifted the restriction on mobility (Fig. 6c). Some cells under flow showed active expansion and contraction at the edges, but the position of the nucleus remained fixed. We suggest that this lack of migration is the result of reduced actin driven traction forces since the basal stress fibers disappeared and F-actin is peripheral under flow condition.

## Discussion

Tubular fluid flow in the kidney causes drag on the apical surface of epithelial cells and these forces modulate cytoskeletal stress. This in turn activates multiple intracellular signaling pathways causing reorganization of cytoskeleton and cell remodeling. Our experiments allowed us to dissect the reorganization into several distinct stages (Fig. 2a). The process seems to involve a Ca^2+^ influx through MSCs.

The initial decrease in cytoskeletal stresses is consistent with our previous findings that sudden change in shear stress causes reversible compression in α-actinin. Repetitive application results in progressive adaptation [25]. The shear stress amplitude in this study is an order of magnitude smaller than in our previous study indicating that we are far from saturation in these experiments. F-actin shows no significant change within this time suggesting that this process only involves the conformational changes of the linking proteins or deformation of actinin itself (Fig. 2e). This adaptation to mechanical stress has also been observed with magnetic beads attached to the cell surface [16].

The shear-induced increase in cytoskeletal stress and corresponding inward contraction of F-actin with time appears to be an essential process for reorganization of the cytoskeleton since this is followed by extended reduction in cytoskeletal stresses and redistribution of F-actin to the periphery. Similar increase in cytoskeletal stress (as in Phase-II) has also been observed using magnetic beads at early stages of cell adaptation [16]. Recently, Martin, *et. al.* reported that pulsed apical contractions powered by actomyosin pull the cytoplasm inward towards the nucleus [36]. This contractile force is known to facilitate actin polymerization via actin binding proteins at focal adhesions [37]. Thus, the observed inward movement of actin during phase II (Fig. 2e,g) indicates the contraction of actin network that triggers the subsequent actin reassembly. Our results thus suggest that such actin-myosin contraction can be activated by flow shear stress so that the net effect of fluid shear stress acts via body stress in the cell.

Long term adaptation seems to involve Rho GTPases and its downstream targets. Rho-associated kinase (ROCK) is a key regulator for cytoskeleton reorganization, focal adhesion development and cell motility [38], [39], [40]. In MDCK cells, application of 20 µM Y27632, a Rho-ROCK inhibitor, caused disassociation of F-actin, and blocked the cytoskeleton remodeling in flow (Fig. S2). Our results suggest Rho GTPases may participate in flow induced actin reorganization. It has been reported that applying a mechanical force to the cell surface of MDCK cells leads to Rho translocation [19], and Rho-kinase regulates myosin light chain phosphorylation that increases cell contractility and alters the formation of actin stress fibers [19], [39]. The observed contractile force in phase-II (Fig. 2d,e) seems to be the result of Rho mediated myosin contraction that facilitates the actin restructuring. In addition, flow-induced Rho activation facilitates cytoskeleton reorganization in endothelial cells [19], [27], [41], [42], [43] with an initial transient inactivation that is followed by an increase peaking at ∼60 minutes [9]. Although this time scale (in the range of 5–60 min) is similar to our observation for actin disassociation and reassembly under flow (Fig. 2e), the cytoskeletal dynamics are different for endothelial and epithelial cells probably because they have different cytoskeletal prestress and organization [5].

The whole adaptation process can be initiated by MSCs since changes in structure and motility were blocked by Gd^3+^ and GsMTx4. A similar effect has been reported with Gd^3+^ for stresses from magnetic beads [16]. Flow-induced MSC activation and Ca^2+^ transients have been observed in MDCK cells [34], [44]. We have also shown that shear stress causes a transient Ca^2+^ response in MDCK cells and the Ca^2+^ influx can be blocked by MSC inhibitors Gd^3+^ and GsMTx4 (Fig. S1). The time dependence of the Ca^2+^ response shows that intracellular Ca^2+^ peaked within 1 min in flow, preceding the contractile actin ring formation. These observations suggest a connection between Ca^2+^ increase and downstream signaling pathways, such as Rho GTPases activation. The reorganization, however, does not require a continued elevation of Ca^2+^, but the transient elevation appears to lead to activation of a long lived later messenger such as Rho or possibly altered prestress in the cytoskeleton.

**Figure 7:**
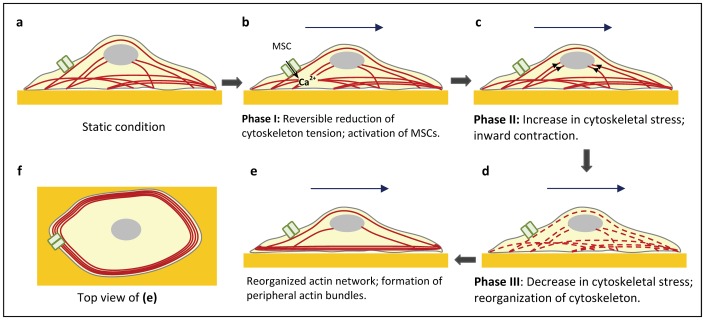
Schematic of proposed multiphase-adaptation to fluid shear stress in epithelial cells.

In conclusion, fluid shear stress regulates cytoskeletal dynamics triggered by Ca^2+^ permeable MSCs and progressing through a series of structural adaptations, leading to a reduction of cytoskeletal tension. The process is illustrated schematically in Fig. 7. This remodeling is important for the cell to minimize the stress that it experiences and/or to optimize its structure to resist the external force so as to minimize the internal stress gradients.

## Materials and Methods

### Actinin-sstFRET sensor construction

The sstFRET stress sensor was constructed with Cerulean (a CFP) as the donor, Venus (an improved version of YFP) as the acceptor, and a spectrin repeat domain as the linker that was subcloned from non-erythrocytic spectrin isoform 1 [29], Fig. 1a. To insert sstFRET into α-actinin, we subcloned α-actinin into pEYFP-C1 vector (Clontech, Mountain View, CA, USA) by replacing the original YFP gene. The new construct was called PEG-actinin. The sstFRET was then inserted into PEG-actinin to get PEG-actinin-sstFRET. For maximum sensitivity, sstFRET is inserted into the middle of actinin, at amino acid 300; further details of the construction are given elsewhere [29]. The sstFRET stress sensors have previously been characterized for their stress sensitivity and it has also been shown that the sensors have no adverse effect on the host protein [29].

### Microfluidic chamber and environmental control

The microfluidic chip consists of a PDMS flow channel that is 500 µm wide, 100 µm high, and 10 mm long. The flow channel is held between two parallel glass plates; a glass cover slip base and a glass top. Holes through the PDMS form the inlet and outlet of the channel. For long-term imaging under flow, the microfluidic chip was placed in an incubator (INUB-ZILCSD-F1-LU, Tokai Hit CO., Ltd, Japan) at 37°C and 5% CO_2_. Cell culture media was perfused through the chamber using a syringe pump (Harvard Apparatus PHD2000). The fluid shear stress (*τ*) was calculated using the relationship *τ = 6Qμ/wh^2^*, where *Q* is the flow rate, *μ* is the dynamic viscosity of the perfusing media, *w* and *h* are the width and the height of the channel, respectively; a viscosity value of 0.8×10^−3^ Pa·s was used [45].

### Cell culture

MDCK cells (ATCC) were cultured directly in the microfluidic chamber. Cells were cultured in Dulbecco's Modified Eagle Medium (DMEM) having 10% fetal bovine serum, with 1% penicillin and streptomycin. To seed the cells in the flow chamber, the microfluidic chamber were first coated with fibronectin and then the media with suspended cells was perfused into the chamber using a pipette. The microfluidic chip was placed in the incubator and culture media was changed every 24 hours. Experiments were conducted after culturing the cells for 3 days with typical confluence of 80–90%. Cells were transfected with sstFRET α-actinin 24 hours before the experiments using FuGENE 6 transfection reagent (3.3∶1, FuGENE 6 volume to DNA weight ratio). Average transfection efficiency was 20–30%. During the experiments, the chamber was perfused with Phenol-red free DMEM (Invitrogen™) in order to eliminate background fluorescence from the media.

### FRET imaging and analysis

Fluorescence imaging was done using a Zeiss inverted microscope with 63× oil immersion objective (Axiovert 200 M, Zeiss). Images were acquired using Hamamatsu EM-CCD camera (ImagEM C9100-13, Hamamatsu, Japan). Both emission wavelengths (CFP and YFP) were acquired simultaneously using a dual-view optical system (Optical Insights, USA) having an excitation filter set that includes a bandpass filter (436±20 nm) and a dichroic mirror (455DCLP), and an emission filter set that includes two emission filters (480±30 nm and 535±40 nm) and a dichroic mirror (505DCXR). Time-lapse images were obtained using Zeiss software (Axiovision, Zeiss). The images from CFP and YFP channels were aligned and processed to obtain the ratio of acceptor to donor (FRET A/D ratio) using Image-J (NIH) software following previously published methods [28], [46]. The bleedthrough of CFP into YFP was measured prior to the experiments and subtracted from YFP images to obtain the true FRET signal. The spatiotemporal variation of the FRET ratio is shown in a 16 color map. The warm colors (red) indicate a higher ratio, i.e., a lower α-actinin stress, while cool colors (blue) indicate a lower FRET ratio (higher α-actinin stress).

### Immunostaining F-actin

Cells were treated with 4% paraformaldehyde at room temperature for 15 minutes and washed three times with phosphate buffered saline (PBS). Cells were then incubated in 0.5% Triton X-100 solution for 15 min and washed three times with PBS. Cells were stained with 0.16 µM phalloidin-Alexa Fluor 568 (Invitrogen) for 20 minutes, washed three times with PBS and were then observed under the microscope.

## Supporting Information

Figure S1 **Ca^2+^ response to flow shear stress.** Time course of intracellular Ca^2+^ response to a stepwise increase in shear stress from 0.03 to 1 dyn/cm^2^ in control (red dots), 100 µM Gd^3+^ (solid squares), and 5 µM GsMTx4 (unfilled squares), showing that Ca^+2^ influx was blocked by MSC inhibitors.
(TIF)

Figure S2 **Effect of Rho-ROCK inhibitor Y27632 on the formation of actin fibers and cytoskeletal stress.** a: Live cell imaging of actinin-sstFRET (CFP channel) during the application of Y27632 at times indicated in (b), showing that blockage of Rho-ROCK reversibly disassociates actin stress fibers. The scale bar represents 10 µm. b: Average FRET ratio over the two cells in (a). c: FRET ratio measured in a cell subjected to shear stress of 0.74 dyn/cm^2^ in the presence of 20 µM Y27632, showing that the inhibitor blocked the flow induced changes in cytoskeleton stresses.
(TIF)

Movie S1 Time-lapse CFP images of actinin-sstFRET expressing MDCK cells in response to a shear stress of 0.74 dyn/cm^2^, showing the shear induced actin reorganization. The arrow (top left) indicates the start of flow. The nucleus ‘ring formation’, few minutes after application of shear, and later peripheral actin arrangement is readily observable. The images were captured every 2 min for a total of 3 hours.
(AVI)

Movie S2 FRET response of MDCK cells to a shear stress of 0.74 dyn/cm^2^, showing shear induced FRET changes and a net increase in FRET ratio upon the application of shear. The arrow indicates the start of flow. The images were captured every 2 min for 3 hours.
(AVI)

Movie S3 Time-lapse CFP images of actinin-sstFRET expressing MDCK cell in static environment. Actin dynamics that were observed in cells under shear are absent and the cell is mobile. The images were taken every 3 min for 3 hours.
(AVI)

Movie S4 Typical FRET changes in MDCK cell under static condition showing only slight variations with time. The images were captured every 3 min for a total of 3 hours.
(AVI)

Movie S5 Time-lapse CFP images of actinin-sstFRET transfected MDCK cell in response to a shear stress of 0.74 dyn/cm^2^ in the presence of 100 µM Gd^3+^, showing no significant redistribution of actin due to shear. The arrow (top left) indicates the onset of flow. The images were taken every 2 min for a total of 3 hours.
(AVI)

Movie S6 FRET response of MDCK cell to a shear stress of 0.74 dyn/cm^2^ in presence of 100 µM Gd^3+^, showing only small variations and no apparent change in FRET. The arrow indicates the start of flow. The images were taken every 2 min for a total of 3 hours.
(AVI)
