# Supplementary figures and images for: Interplay between Cytoskeletal Stresses and Cell Adaptation under Chronic Flow
Source: PLoS One. 2012 Sep 19;7(9):e44167. doi: 10.1371/journal.pone.0044167 (PMC3446919; doi:10.1371/journal.pone.0044167)

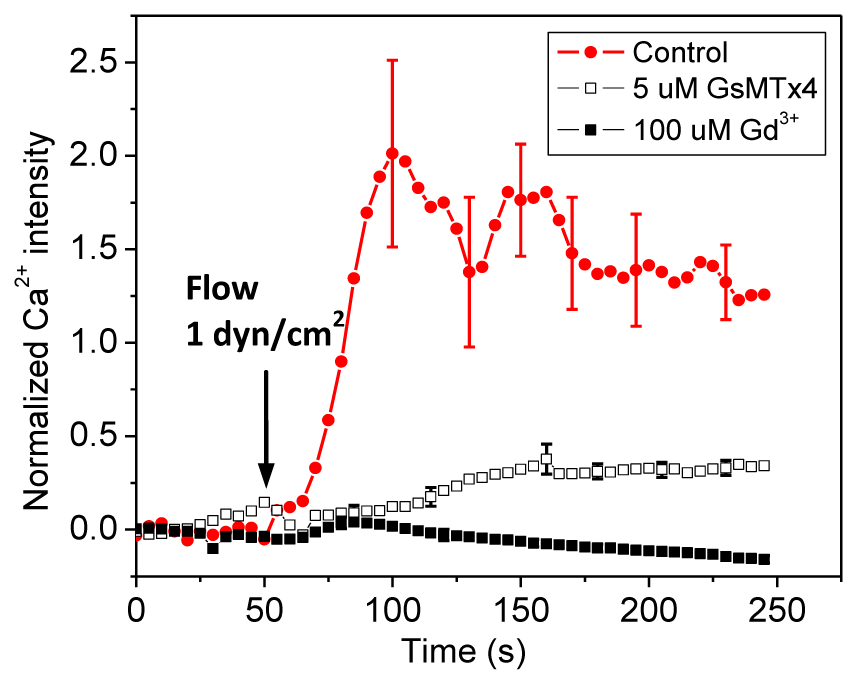

Supplement: Figure S1 — Ca2+ response to flow shear stress. Time course of intracellular Ca2+ response to a stepwise increase in shear stress from 0.03 to 1 dyn/cm2 in control (red dots), 100 µM Gd3+ (solid squares), and 5 µM GsMTx4 (unfilled squares), showing that Ca+2 influx was blocked by MSC inhibitors. (TIF) [file pone.0044167.s001.tif]

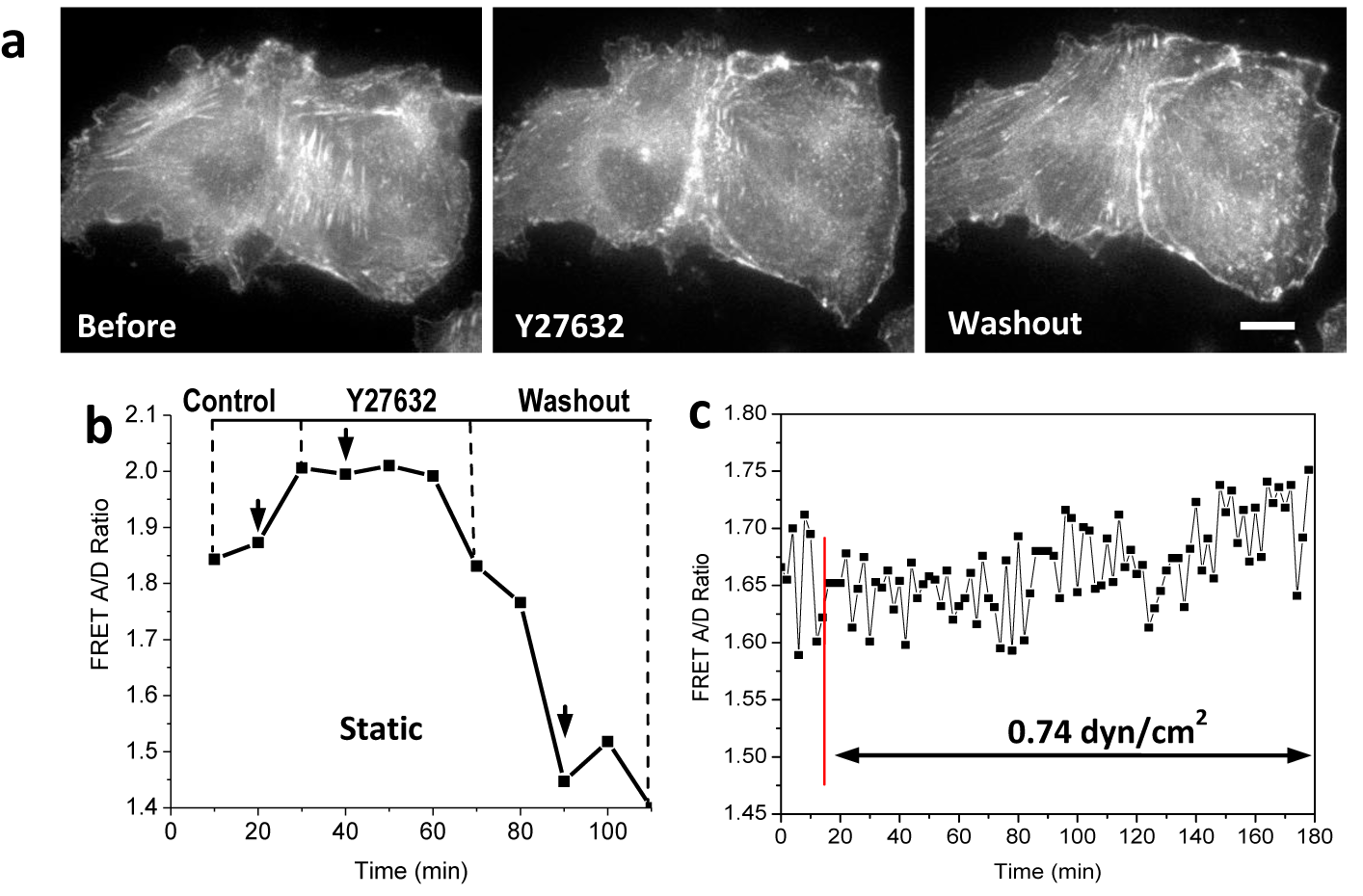

Supplement: Figure S2 — Effect of Rho-ROCK inhibitor Y27632 on the formation of actin fibers and cytoskeletal stress. a: Live cell imaging of actinin-sstFRET (CFP channel) during the application of Y27632 at times indicated in (b), showing that blockage of Rho-ROCK reversibly disassociates actin stress fibers. The scale bar represents 10 µm. b: Average FRET ratio over the two cells in (a). c: FRET ratio measured in a cell subjected to shear stress of 0.74 dyn/cm2 in the presence of 20 µM Y27632, showing that the inhibitor blocked the flow induced changes in cytoskeleton stresses. (TIF) [file pone.0044167.s002.tif]
